# Dental Expression and Role in Palliative Treatment

**DOI:** 10.4103/0973-1075.53508

**Published:** 2009

**Authors:** Rajiv Saini, PP Marawar, Sujata Shete, Santosh Saini, Ameet Mani

**Affiliations:** 1 Department of Periodontology, Periodontology and Oral Implantology, Rural Dental College-Loni, Maharashtra, India; 2 Department of Periodontology, Microbiology, Rural Dental College-Loni, Maharashtra, India

**Keywords:** Dental expression, Hospice care, Oral lesions, Pain, Palliative care

## Abstract

World Health Organization defines palliative care as the active total care of patients whose disease is not responding to curative treatment. Palliative care for the terminally ill is based on a multidimensional approach to provide whole-person comfort care while maintaining optimal function; dental care plays an important role in this multidisciplinary approach. The aim of the present study is to review significance of dentist's role to determine whether mouth care was effectively assessed and implemented in the palliative care setting. The oral problems experienced by the hospice head and neck patient clearly affect the quality of his or her remaining life. Dentist plays an essential role in palliative care by the maintenance of oral hygiene; dental examination may identify and cure opportunistic infections and dental disease like caries, periodontal disease, oral mucosal problems or prosthetic requirement. Oral care may reduce not only the microbial load of the mouth but the risk for pain and oral infection as well. This multidisciplinary approach to palliative care, including a dentist, may reduce the oral debilities that influence the patient's ability to speak, eat or swallow. This review highlighted that without effective assessment of the mouth, the appropriate implementation of care will not be delivered. Palliative dental care has been fundamental in management of patients with active, progressive, far-advanced disease in which the oral cavity has been compromised either by the disease directly or by its treatment; the focus of care is quality of life.

## INTRODUCTION

World Health Organization define palliative care as the active total care of the patient whose disease is not responsive to curative treatment. Control of pain, other symptoms, and psychological, social and spiritual problems is paramount. The goal of palliative treatment is the achievement of the best possible quality of life for patients and their families.[1] The treatment should focus on the improvement of the quality of life instead of straining curative treatment approach. In palliative medicine, an interdisciplinary approach is inevitable and essential. The importance of dental care is often overlooked due to the omission of the dentist as a member of the palliative care team.[2] Lesions of the oral cavity have an immense impact on the quality of life of patient with complex advanced diseases. They caused considerable morbidity and diminish patients physical and psychological well being. The consequences of an unhealthy or painful oral cavity are significant. Not only are there physical implications of reduced oral intake and weight loss but, in addition, there may be psychological effects due to impaired communication and feelings of exclusion and social isolation. Good oral hygiene is fundamental for oral integrity as it greatly affects the quality of life.

## DENTAL REFLECTION IN PALLIATIVE CARE

Dentist proficiency play a major role in palliative care and treatment. Traditional oral hygiene care may not be appropriate for residents who are acutely sick, unconscious, non-responsive, or terminally ill. Palliative oral care focuses on strategies for maintaining resident quality of life and mouth comfort. Oral health goals of palliative care includes quality care, free of pain and infection, individual is comfortable, mouth moist and clear from dental plaque, calculus or food debris. As oral lesions are indicators of disease, progression and oral cavity can be a window to over all health. Dental expression in palliative care may be defined as the extended dental services with a central goal of providing preeminent feasible oral care to terminally ill or far advanced diseased patients, where oral lesions or conditions greatly impact on the quality of remaining life of patients, the initiation and progression of oral lesions may be related to direct or indirect succession of disease, its treatment or both.

## ORAL LESIONS: THE CLINICAL PICTURE

Oral problems in palliative patients may be related to, (a) direct effect of the primary disease, (b) indirect effect of the primary disease, (c) treatment of the primary disease, (d) direct/indirect effect of a coexisting disease, (e) treatment of coexisting disease, (f) combination of the above factors.[3] The assessment of oral problem is essentially similar to assessment of other medical problems. It involves taking a history, performing an examination, and the use of appropriate investigations, the oral examination involves general observation, intra-oral examination, and extra-oral examination[4] that include the examination of lips and gums, teeth, cheek, floor and roof of mouth, and lymph nodes. Oral symptoms are common in palliative care patients[3,5–8] [Table 1].

**Table 1:** Prevalence of oral symptoms in palliative care patients

Study	Population type/size	Dry mouth	Oral discomfort	Taste disturbance	Difficulty in chewing	Difficulty in swallowing	Difficulty in speaking
Gordon *et al*.[5] (1985)	Hospice inpatients (N = 31)	62	55	31	52	Not detected	59
Aldred *et al*.[6] (1991)	Hospice inpatients (N = 20)	58	42	26	Not detected	37	Not detected
Jobbins *et al*.[7] (1992)	Hospice inpatients (N = 197)	77	33	37	Not detected	35	Not detected
Davies[8] (2004)	Hospice support team patients (N = 120)	78	46	44	23	23	31

## ORAL CARE IN PALLIATIVE CARE

The basic principle of oral care in palliative care (OCPC) is focused primarily on the principle that good oral hygiene is the fundamental for oral integrity. Early clinical diagnosis of the oral lesions or conditions in the palliative patients should be done and appropriate actions must be instituted to minimize pain and suffering by giving the symptomatic relief. Systematic assessment is essential, using a glove, torch and tongue depressor, and removing any dentures. The causes of oral lesions may be fungal, viral, bacterial, ulcerative, immunosupression, radiation, lack of oral hygiene, and so on. Most patients have at least one symptom, many patients have several.[6,8] Oral infections are also common in palliative care patients. There have been number of studies that have looked at oral candidosis in this group.[5,6] Active dental caries have been reported in 20-35 percent of patients[6,7] and active gingivitis in 36 percent patients.[5] The common oral problems in palliative patients includes, xerostomia (dry mouth), sore mouth, thrush, swallowing problems, sore lips, tastes/odor, soreness under dentures, heavy mucous, difficulty in speaking and pain from one or more of these problems [Table 2].

**Table 2:** Common oral problems in palliative patients

Oral lesion/condition	Features	Causes
Xerostomia	Dry mouth	Anxiety, and depression
	Coated tongue	Drugs (side effects): Antimuscarinics, diuretics
	Tongue may appear glossy	Mouth breathing, un-humidified oxygen, infection
	Salivary gland hypo function	Dehydration, restricted diet/fluid intake
		Surgery, chemotherapy or radiotherapy to the head and neck region
		Injury to salivary glands or buccal mucosa
		Hypothyroidism, Autoimmune disease like Sarcoidosis, Jorgen's syndrome and Alzheimer's disease etc.
Oral candidiasis	Creamy white patches	Prolonged antibiotics
	Multiple white to yellow soft plaques	Diabetes mellitus
	Areas may bleed and burn	Impaired immunity (e.g. chemotherapy/radiotherapy)
	Taste alterations	Dry mouth
	Usually accompanied by xerostomia	Prolonged wearing of dentures
Angular cheilitis	Cracking, fissuring, irritation with red areas at mouth corners	A fungal or bacterial infection most often associated with denture stomatits
	Painful mouth opening	A vitamin B deficiency
Denture stomatits	Generalized redness in upper palate (rarely lower palate)	Denture not cleaned properly and dentures that remain in mouth for longer period of time
	Chronic irritation and redness	A fungal or bacterial infection
	Mostly asymptomatic but sometimes painful and may bleed	
Mucositis	Inflammation and bleeding of the oral soft tissues of lips, cheeks, gums, and tongue	Mucositis is mouth pain that develops due to the break down of oral tissues
	Pain, nutritional problems, and increase risk of infections	
Dysphagia	Inability to hold or control food	Weakened musculature and control over facial muscles and tongue
	Pocketing of food	Sensation loss
	Incompetent lips	Patient continually lie in a flat or reclined position
	Higher risk for choking food etc.	
Ulceration	Apthous ulcers (canker sores)	Medications
	Crater type sore or mucous membrane	Nutritional deficiency
	Painful	Stress
	Interference with speech and swallowing	Acidic food
	Sometimes pus formation	Trauma
Taste disorders	Taste alterations	Depression
	Decreased taste sensitivity	Head and neck radiotherapy
	Sometimes burning sensation	Medications for diseases like diabetes, depression, anti Parkinson, seizures etc.
Sore/dry lips	Lip tissues are flaking and rough	Dehydration of lips and pores blockage

Mouth care is considered one of the most basic of nursing activities, and palliative care patients are especially vulnerable to oral problems.[9] The management of oral problems or lesions in palliative patients should be carried out as a team work and definite treatment protocol should be followed by both non-dentist palliative care physician and by dental expert [Table 3] and it is strongly marked that palliative care is a multidisciplinary approach and role of dentist is essential to maintain optimal oral health. In addition to the treatment of symptomatic clinical lesions in oral cavity, an essential oral care protocol to be undertaken that emphasis on routine oral examination and care of palliative patients. The recommendation for routine oral health includes use a ultra soft brand of toothbrush (as hard toothbrushes may lead to abrasions), toothpaste should only be used when an individual is able to spit and swallow as tooth paste can burn sensitive oral tissues and foaming action can induce gag reflex and may lead to choking. Mouthwashes with alcohol and petroleum based products for lip care should not be used. Dentures should be removed and soaked overnight in dilute sodium hypochlorite or chlorhexidine gluconate 0.2% depending upon its material.

**Table 3:** Management of common oral problems in palliative patients

Oral lesion/condition	Non-dentist palliative care physician	Dentist-role and expertise
Xerostomia	Review medication	Specialized oral hygiene to remove coating or plaque by
	Oral care is encouraged	dental hygienist or dentist
	Maintain hydration by regular, cold unsweetened drinks	Salivary substitutes or oral balance gel
	Ice to suck or sugar free chewing gum	Chlorhexidine gluconate 0.2%, mouthwash used twice daily for 1 min. Dilute1:1 with water if too strong
	Use of atomized water spray	
Oral candidiasis	Nystatin suspension 1 ml, as a mouthwash then swallowed, 4 times daily for 7-14 days	Chlorhexidine gluconate 0.2%, mouthwash 10 ml twice daily
	Fluconazole 50-100mg daily for 7-10 days if topical antifungal are ineffective	Dentures to be examined and cleaned thoroughly
	If angular cheilitis present Nystatin cream or Miconazole Gel topically 4 times a day	Scaling and polishing of teeth
	For persistent infection further investigation will be required	
Angular cheilitis	Antifungal agent or antibacterial agent	Clan and fit dentures and dental prosthesis
	Nystatin suspension, or miconazole gel (topically 4 times daily)	
	Multivitamin supplementations	
Denture stomatits	Eased by using an anti-fungal agent or antibacterial agent	Realignment of dentures and dry mouth product (Mucco, Biotene, KY gel) can be placed under dentures for comfort
	Keep dentures clean by scrubbing and then soak dentures daily in solution of ½ water to ½ vinegar	Professionally cleaning and polishing of denture
Mucositis	If painful mucositis, benzydamine hydrochloride 0.15% (Difflam) 15 ml 2-3 hourly for up to 7 days. Dilute 1:1 with water if stings.	Dental prosthesis to be removed and thoroughly cleaned and rectified of any technical error
	For analgesia: soluble paracetamol gargle	Mouth washes to be administered as per need and clinical picture of the lesions
	Consideration of co-codamol or morphine – if more severe pain	
Dysphagia	Head of the bed to be raised for ease of the patient	Specialized oral hygiene to remove coating or plaque by
	Use of suction machine if available	dental hygienist or dentist
	Removable of debris by gauze	Oral physiotherapy
Ulceration	Identify cause if possible	Correction of ill fitting dentures or dental caries if present
	Chlorhexidine gluconate 0.2% mouthwash twice daily	
	If persistent ulcers, consider sending a swab for culture	
	Treat herpetic ulcers on lips with topical acyclovir; use oral acyclovir for herpes infection in the mouth	
	If ulceration is foul smelling, Metronidazole 400 mg 3 times a day orally	
	If ulcers painful, use benzydamine hydrochloride 0.15% oral rinse, then topical steroid (e.g. hydrocortisone lozenge, triamcinolone in orabase).	
Taste disorders	Treatment follows as of xerostomia	Topical application of analgesia
	Avoid stimulating factors	Scaling and polishing of teeth
Sore/dry lips	Water based lip balms	Consultation from the dentist for diagnosis
		Symptomatic treatment to be followed

## CONCLUSION

The oral cavity has the potential to harbor at least 600 different bacterial species, and in any given patient, more than 150 species may be present, surface of tooth can have as many as a billion bacteria in its attached bacterial plaque.[10] In end-of-life care, examination of the mouth and re-examination of the mouth is a very important task and careful assessment is necessary each day. Oral problems are common complications of cancer treatments, and are highly prevalent in palliative care patients. Oral problems are often overlooked, or perceived as trivial, but causes great distress, pain and discomfort, interfere with appetite, taste, chewing, swallowing, nutrition, speech, social interactions, and sleeping. The palliative care dentist must assess these difficulties, and should focus on the elimination of these problems, appropriate actions must be instituted to at least alleviate symptoms, minimize pain and suffering and provide symptom control. Dental professional are the important members of extended palliative team[11] and they have number of key roles, including (a) training of palliative care professionals, (b) management of complex oral problems, and (c) management of specific oral problems. Increased awareness by all health care professionals and of palliative oral care would go a long way in providing relief, comfort, and consolation to terminally ill patients and their families.
